# Associations of Perceived Classical Chinese Text Reading Ability with Confucian Virtue Cognition and Practice Among Chinese Adolescents: The Indirect Role of Internalization Experience

**DOI:** 10.3390/bs16091659

**Published:** 2026-09-16

**Authors:** Lingrong Shen, Baoling Wu, Bo Yuan

**Affiliations:** 1 Department of Elementary Education, Ningbo University, Ningbo 315211, China; shenlingrong@163.com; 2 Department of Psychology, Ningbo University, Ningbo 315211, China; wbl1792022@163.com

**Keywords:** perceived classical Chinese text reading ability, Confucian virtues, internalization experience, Confucian virtue cognition, Confucian virtue practice, adolescents

## Abstract

Classical Chinese texts are important cultural materials through which adolescents encounter traditional moral values, yet the associations among perceived classical Chinese text reading ability, internalization experience, and Confucian virtue cognition and practice remain insufficiently examined. This study investigated whether junior secondary school students’ perceived classical Chinese text reading ability was associated with their moral cognition and moral practice of the Confucian five virtues: benevolence, righteousness, propriety, wisdom, and integrity. It further examined whether the variables showed a statistical indirect association through internalization experience in traditional cultural learning. Participants were 304 junior secondary school students from Ningbo, China who completed self-developed measures of perceived classical Chinese text reading ability, five-virtue moral cognition, five-virtue moral practice, and internalization experience. Correlation and hierarchical regression analyses showed that perceived reading ability was positively associated with both moral cognition and moral practice after controlling for gender and grade. Cross-sectional mediation analyses identified an indirect association through internalization experience between perceived reading ability and moral cognition, as well as between perceived reading ability and moral practice. However, given the cross-sectional design, these associations cannot establish temporal or causal ordering. These findings suggest that adolescents’ perceived competence in classical Chinese reading is positively associated with Confucian virtue cognition and practice, with internalization experience serving as a plausible intervening process—one that requires longitudinal or experimental evidence to confirm.

## 1. Introduction

Adolescence is a critical period for the consolidation of values, the differentiation of moral judgment, and the gradual translation of moral understanding into everyday behavior (Arnett, 2000; Phinney, 1990). In developmental and educational psychology, considerable attention has been given to adolescents’ academic achievement, problem behaviors, and psychosocial adjustment. Less empirical attention, however, has been paid to how culturally embedded curricular materials may support adolescents’ positive Confucian virtue cognition and practice. This issue is especially important in educational contexts where schooling is expected not only to cultivate academic competence but also to transmit cultural traditions and moral ideals.

In education systems in the Asia-Pacific region, Confucian virtue cognition and practice are often addressed not only through explicit moral education courses but also through culturally embedded materials in language, literature, and history learning. Adolescents may be exposed to traditional moral content in school, but exposure alone does not guarantee meaningful comprehension, value identification, or behavioral enactment. It is therefore important to examine not only whether students encounter traditional cultural texts, but also whether they can understand these texts deeply and connect their meanings with their own moral reasoning and everyday conduct. The present study responds to this issue by treating classical Chinese reading as a psychologically meaningful learning process rather than as a purely linguistic or curricular activity.

In the Chinese cultural tradition, the Confucian five constant virtues—ren, yi, li, zhi, and xin, commonly translated as benevolence, righteousness, propriety, wisdom, and integrity—constitute a central moral framework. Although these virtues are historically rooted, they correspond to psychological concerns that remain relevant to contemporary adolescence, including empathic concern, fairness, rule sensitivity, reflective judgment, trustworthiness, and prosocial responsibility. Understanding how adolescents learn and internalize these virtues can therefore contribute to both cultural psychology and research on Confucian virtue cognition and practice.

Classical Chinese texts are one of the major educational carriers through which traditional moral values are introduced in school curricula. The Chinese Language Curriculum Standards for Compulsory Education emphasize the role of Chinese-language education in cultural inheritance (Ministry of Education of the People’s Republic of China, 2022). From this perspective, reading classical Chinese texts is not merely a matter of linguistic decoding. It also involves understanding historically embedded meanings, interpreting cultural symbols, and identifying the value orientations carried by the text. Because language is closely connected with cultural meaning (Byram, 1997; Kramsch, 2013), students’ ability to understand classical Chinese texts may be closely related to how they comprehend traditional moral concepts.

Classical Chinese texts differ from ordinary reading materials because they often present moral ideas indirectly through condensed language, historical allusions, exemplary figures, and implicit value judgments. Students are therefore required to infer meanings that are not always explicitly stated. For example, understanding a narrative about loyalty, responsibility, or trustworthiness involves more than translating individual words into modern Chinese. It also requires students to reconstruct the social situation, evaluate the intentions and actions of characters, and recognize the moral standard implied by the text. This interpretive process may create opportunities for moral reflection because students are encouraged to compare textual values with their own experiences and social relationships.

In the present study, classical Chinese text reading is conceptualized as a progressively integrated process involving four analytically distinguishable levels. First, linguistic decoding involves recognizing classical vocabulary, function words, syntactic patterns, and sentence-level relations. Second, propositional understanding involves identifying the main idea, reconstructing omitted information, and interpreting or translating expressions in context rather than word by word. These two levels correspond to the construction of surface-level and propositional representations in reading comprehension (Kintsch, 1988). Third, cultural-symbolic interpretation involves situating historical allusions, exemplary figures, ritual concepts, and other culturally embedded symbols within their historical and social contexts. Because textual meaning is constructed within cultural and social contexts, this level requires readers to connect linguistic information with culturally shared knowledge and symbolic meanings (Byram, 1997; Kramsch, 2013). Fourth, value-orientation analysis involves identifying the moral positions implied by characters’ choices, narrative evaluations, and the Confucian virtues represented in the text. This higher-order level requires readers to integrate textual understanding, contextual knowledge, and evaluative interpretation. Previous research on classical Chinese reading has similarly emphasized that successful comprehension extends beyond vocabulary recognition and literal translation to deeper meaning construction and interpretation (Lau, 2019; Lau & Qian, 2025). Although these four levels are theoretically distinguishable, they operate together during comprehension and were not treated as four independent subscales in the present study.

This four-level conceptualization is informed by the construction–integration model of reading comprehension. Within this framework, the relevant process refers to constructing a coherent mental representation of a classical Chinese text: readers decode linguistic forms, build propositional meaning, integrate explicit textual information with relevant historical and cultural knowledge, infer implicit meanings, and evaluate the value orientations embedded in the text. According to the construction–integration model, reading involves multiple levels of processing, from surface-level decoding and propositional understanding to the construction of a situation model (Kintsch, 1988). For classical Chinese texts, this process may place substantial demands on readers’ working-memory resources because students must simultaneously process unfamiliar vocabulary, syntax, rhetorical forms, textual structures, and culturally embedded meanings (Baddeley, 2012; Sweller, 2010). Accordingly, higher-order classical Chinese reading competence involves the integration of linguistic comprehension, textual interpretation, contextual knowledge, and value analysis.

In the present study, this theoretical competence was operationalized as perceived classical Chinese text reading ability because it was assessed through students’ self-reports rather than an objective reading-performance test. The four-item scale sampled students’ perceived ability in vocabulary understanding, main-idea identification, contextual translation, and analysis of characters’ moral qualities. It was therefore intended to index students’ overall perceived competence rather than separately estimate each theoretical processing level.

This perceived reading ability may be relevant to adolescents’ traditional Confucian virtue cognition and practice. Accordingly, higher-order classical Chinese reading competence denotes the theoretical construct, whereas perceived classical Chinese text reading ability denotes its self-reported operationalization in the present study.

This perceived reading ability may be relevant to adolescents’ Confucian virtue acquisition. In the present study, Confucian virtue acquisition refers to adolescents’ current understanding and reported enactment of the Confucian five constant virtues—benevolence, righteousness, propriety, wisdom, and integrity. It is operationalized through two related but distinct domains. Confucian virtue cognition refers to understanding the meanings and normative implications of these virtues, whereas Confucian virtue practice refers to the reported enactment of the virtues in everyday situations. Because moral understanding does not necessarily translate directly into moral action, the two domains were examined separately (Blasi, 1980). The term acquisition is used as an umbrella description of students’ current levels of virtue understanding and enactment; it does not denote developmental stages, a transition from cognition to behavior, or change over time.

Classical texts often include historical figures, ethical choices, and value judgments that may serve as symbolic models for moral learning (Bandura, 1986). In addition, narrative engagement may make readers more receptive to values embedded in texts (Green & Brock, 2000), while cultural psychology emphasizes that language and texts mediate the transmission of cultural meanings (Vygotsky, 1978).

Based on this reasoning, stronger perceived classical Chinese text reading ability may help students understand the situations, characters, choices, and value judgments embedded in classical texts and may thereby support both moral understanding and the behavioral application of moral values. Therefore, the present study proposed the following hypothesis:

**Hypothesis** **1.** *Junior secondary school students’ Perceived Classical Chinese Text reading ability is positively associated with their Confucian virtue cognition and Confucian virtue practice.*

However, the association between perceived classical Chinese text reading ability and moral outcomes is unlikely to occur automatically. The moral meanings embedded in texts need to be understood, experienced as personally meaningful, and integrated into students’ own value systems. Self-determination theory and organismic integration theory suggest that external values and norms become more stable and self-endorsed when individuals understand, accept, and integrate them into the self (Deci & Ryan, 2000; Ryan & Deci, 2017). In educational settings, this process of internalization helps explain why exposure to value-laden content may lead to deeper learning and more stable motivational or behavioral outcomes (Niemiec & Ryan, 2009; Reeve, 2012; Y. Wang et al., 2024). In language learning, autonomous motivation is also related to achievement (Alamer et al., 2025).

In the context of classical Chinese learning, internalization experience refers to students’ perceived meaningfulness, value identification, emotional engagement, and self-integration during the learning of traditional cultural texts. Students with stronger classical Chinese perceived reading ability may be more capable of moving beyond mechanical word-by-word decoding. They may experience lower cognitive load when processing difficult texts and may be better able to grasp the central ideas, moral implications, and cultural spirit of the material. This deeper understanding may make it easier for them to connect traditional values with their own experiences, social relationships, and personal development.

These four aspects represent complementary experiential components rather than a fixed developmental sequence. Perceived meaningfulness concerns the extent to which students regard the moral meanings of a text as significant and relevant to their own experiences. Students who can comprehend the plot, historical context, and implicit value orientation of a classical text may be better able to connect otherwise abstract moral propositions with situations in everyday life. Educational applications of self-determination theory similarly suggest that personally meaningful rationales and perceived relevance are associated with more autonomous engagement in learning (Niemiec & Ryan, 2009; Reeve, 2012).

Value identification refers to recognizing and personally endorsing the worth of the virtue conveyed by a text, rather than merely treating it as an externally imposed curricular requirement. This aspect corresponds to identified regulation in organismic integration theory, in which individuals consciously accept a value as personally important (Deci & Ryan, 2000; Ryan & Deci, 2017). Stronger perceived classical Chinese text reading ability may give students greater interpretive access to characters’ motives, choices, consequences, and moral judgments. Such access may be associated with clearer Confucian virtue cognition and a stronger willingness to regard the textual values as personally worthwhile.

Emotional engagement denotes affective involvement with the characters, moral conflicts, and consequences represented in a text. Research on narrative transportation indicates that readers who become absorbed in a narrative may be more receptive to its implications (Green & Brock, 2000). When adolescents understand a classical text’s situation and implicit meaning, they may be more likely to experience empathy, admiration, disapproval, or moral concern. These emotional responses may make virtue concepts more salient and may be associated with the motivational connection between understanding a virtue and reporting its enactment in daily life.

Self-integration refers to connecting an endorsed virtue with one’s self-concept, personal goals, and standards for action. Within self-determination theory, integration occurs when an accepted regulation is brought into coherence with other values and aspects of the self (Deci & Ryan, 2000; Ryan & Deci, 2017). A coherent interpretation of a classical text may provide a basis for relating its moral message to one’s own identity, while stronger self-integration may be associated with both more elaborated Confucian virtue cognition and more consistent reported virtue practice. Taken together, the four aspects clarify why internalization experience was hypothesized as a statistical link between perceived classical Chinese text reading ability and Confucian virtue acquisition. They are not treated as a temporal chain in the present cross-sectional study.

Internalization experience may, in turn, be associated with stronger Confucian virtue cognition and moral practice. When students experience classical texts as meaningful and personally relevant, the values conveyed by those texts may become more than external curricular knowledge. They may become self-endorsed standards for interpreting social situations and guiding behavior. In this sense, internalization experience may be statistically involved in the association between perceived classical Chinese text reading ability and both understanding and practicing the Confucian five virtues. Therefore, the present study proposed the following hypothesis:

**Hypothesis** **2.** *Perceived Classical Chinese Text Reading Ability is indirectly associated with students’ Confucian virtue cognition and practice through internalization experience in traditional cultural learning.*

Gender and grade may also be relevant when examining these associations. Research on adolescent reading has documented grade- and gender-related differences in reported reading-comprehension strategy use (Denton et al., 2015), while longitudinal research indicates that prosocial behavior and its relations with empathy may vary across adolescence and may follow different patterns for boys and girls (Van der Graaff et al., 2018). These findings do not establish that gender or grade necessarily changes the association between perceived classical Chinese text reading ability and Confucian virtue acquisition, particularly in the present cultural context. They nevertheless justify controlling for gender and grade when estimating the focal associations and conducting supplementary interaction tests to examine whether those associations appear stable across demographic groups. Because direct prior evidence for directional moderation in this specific context was limited, no formal moderation hypothesis was proposed; the moderation analyses were explicitly exploratory and were not part of the two primary hypotheses.

Although theoretical and educational discussions have long suggested that classical Chinese learning may have a moral-cultivating function, several empirical gaps remain. First, few studies have treated perceived classical Chinese text reading ability as a measurable cognitive variable rather than as general exposure to traditional culture. Second, relatively little research has distinguished between adolescents’ cognition of traditional moral values and their tendency to practice these values in daily life. Third, the psychological mechanism linking text comprehension with moral outcomes has not been sufficiently tested. To address these gaps, the present study examined the associations among perceived classical Chinese text reading ability, internalization experience in traditional cultural learning, and traditional Confucian virtue cognition and practice among junior secondary school students. The study contributes to research on reading comprehension, cultural learning, and Confucian virtue cognition and practice by conceptualizing classical Chinese reading as a process of meaning construction and value interpretation. Given the cross-sectional design, the proposed mediation model was evaluated as an associational model and not as evidence of temporal or causal ordering.

The theoretical novelty of this study lies in integrating three bodies of literature that have often been examined separately. First, it extends the construction-integration perspective from literal or propositional comprehension to the interpretation of culturally embedded value orientations in classical Chinese texts (Kintsch, 1988). Second, it contributes to moral-education research by distinguishing Confucian virtue cognition from reported Confucian virtue practice, rather than assuming that curricular exposure or moral understanding is equivalent to behavioral enactment (Blasi, 1980). Third, it applies self-determination theory and organismic integration theory to traditional cultural-text learning by examining whether perceived meaningfulness, value identification, emotional engagement, and self-integration jointly constitute an internalization experience associated with both virtue outcomes (Deci & Ryan, 2000; Ryan & Deci, 2017). The proposed model therefore connects cognitive access to textual meaning with motivational endorsement of textual values. Its contribution is this cross-domain integration and its initial empirical examination in adolescent classical Chinese learning, rather than a claim to establish a new causal or developmental sequence.

## 2. Materials and Methods

### 2.1. Participants and Procedure

Participants were recruited through purposive sampling from three public junior secondary schools in Ningbo, Zhejiang Province, China. The purposive selection was conducted at the school level. Two explicit criteria were used to obtain variation in educational context: administrative location (urban versus township) and locally recognized school type (key versus ordinary). Accordingly, the study included one key urban school, one ordinary urban school, and one township school. After the schools agreed to participate, students were recruited from intact classes rather than selected according to individual family characteristics. A total of 320 questionnaires were distributed, and 304 valid responses were obtained, yielding a valid response rate of 95.0%. The available individual-level background variables were age, gender, and grade. Parental education, parental occupation, household income, and other indicators of family socioeconomic background were not collected; therefore, the sample should be described as varied in observed school context, not as demonstrating maximum variation in family background (Patton, 2015).

Participants ranged in age from 13 to 15 years (*M* = 13.85, *SD* = 0.63). The sample included 162 boys and 142 girls. In terms of grade level, 87 students were in Grade 7, 176 were in Grade 8, and 41 were in Grade 9. Data were collected in intact classes after consent procedures were completed. Participation was voluntary, and students were informed that they could withdraw from the study at any time. Informed consent was obtained from the participating students and their legal guardians. Ethics approval for the study was granted by the Ethics Committee of the Department of Psychology and Institute of Psychology, Ningbo University on 18 March 2023.

### 2.2. Measures

Because established instruments that simultaneously assess perceived classical Chinese text reading ability, Confucian virtue cognition and practice, and internalization experience are limited, the core measures were developed for this study. Instrument development proceeded in four stages. First, operational definitions were derived from the reading-comprehension, Confucian-virtue, and internalization frameworks described in the Introduction, together with the curricular content of classical Chinese learning. Second, an expert panel comprising three experienced frontline Chinese-language teachers and one educational psychology specialist reviewed the items for construct relevance, content coverage, age-appropriate wording, and clarity; wording was revised in response to this review. Third, item discrimination, internal-consistency reliability, and exploratory factor analysis were examined after data collection. Fourth, confirmatory factor analysis was conducted to evaluate the hypothesized four-factor measurement structure. With the exception of demographic information, all items were rated on a 7-point Likert scale ranging from 1 (completely inconsistent/unable) to 7 (completely consistent/able), with higher scores indicating higher levels of the corresponding overall construct. Because no independent pilot or validation sample was available, the psychometric evidence reported here should be regarded as preliminary.

Perceived Classical Chinese Text reading ability. This four-item self-report scale assessed students’ perceived overall competence in classical Chinese text reading. The items sampled four aspects of reading performance: vocabulary understanding, main-idea identification, contextual translation, and analysis of characters’ moral qualities. These aspects corresponded to progressively deeper forms of text processing; however, the four items were not intended to constitute four independent subscales. Instead, their mean score was used as an overall indicator of students’ perceived classical Chinese text reading ability. Because the instrument relied on self-reports, it reflected students’ perceptions of their reading competence rather than their objectively demonstrated reading performance.

Confucian virtue acquisition: Cognition and practice. This scale assessed two related domains of adolescents’ Confucian virtue acquisition. The cognition subscale assessed students’ understanding of the meanings and normative implications of benevolence, righteousness, propriety, wisdom, and integrity. The practice subscale assessed the frequency with which students reported enacting behaviors corresponding to these virtues in everyday contexts. These subscale scores represent current levels of virtue understanding and reported practice rather than developmental stages or change over time. After one item with weak discrimination was removed, the final scale contained five cognition items and five practice items.

Internalization experience in traditional cultural learning. This four-item scale assessed overall internalization experience by sampling four theoretically related facets: perceived meaningfulness, or experiencing the text’s moral message as personally significant and relevant; value identification, or recognizing and endorsing the value of the virtues conveyed by the text; emotional engagement, or experiencing affective involvement with the text’s characters, conflicts, and moral implications; and self-integration, or connecting the endorsed virtues with one’s self-concept and standards for action. Because the four items were designed to form a brief overall measure and exploratory factor analysis supported a one-factor structure, the items were combined to represent overall internalization experience; facet-specific subscale scores were not calculated or interpreted.

The brief measures followed a content-sampling approach. The four reading items were indicators of one overall perceived reading-ability construct, rather than four independent constructs. Likewise, the five cognition items and five practice items sampled the five Confucian virtues within two broader domains, and the four internalization items sampled related experiences within one overall internalization construct. Thus, the analyses used overall scale or subscale scores and did not estimate vocabulary understanding, main-idea identification, contextual translation, individual virtues, or the four internalization facets as separate single-item constructs. This scoring decision does not remove the limitation that each specific content facet was represented by only one item; it restricts interpretation to the broader constructs.

Demographic variables. Students reported their gender and grade level. Gender and grade were included as control variables in the main analyses and as exploratory moderators in supplementary analyses. Grade 7 served as the reference group in regression models. This distinction was made to keep the primary analyses focused on the two stated hypotheses: gender and grade were covariates in the hierarchical regressions, whereas their interaction terms were examined only as supplementary robustness checks.

### 2.3. Data Analysis

Descriptive, reliability, correlation, regression, mediation, and moderation analyses were conducted using IBM SPSS Statistics for Windows, Version 27.0 (IBM Corp., Armonk, NY, USA), following standard procedures for behavioral science research (Field, 2018). The unmeasured latent method-factor models were estimated in Python 3.12 using normal-theory maximum likelihood. Data screening, item analysis, internal-consistency reliability analysis, and exploratory factor analysis (EFA) were first conducted to evaluate the measures. Descriptive statistics and Pearson correlations were then calculated for the main variables. Hierarchical regression analyses were used to examine whether perceived classical Chinese text reading ability predicted moral cognition and moral practice after controlling for gender and grade.

For the confirmatory factor analysis (CFA), the 18 retained focal items were specified to load on four correlated latent factors: perceived classical Chinese text reading ability (4 items), Confucian virtue cognition (5 items), Confucian virtue practice (5 items), and internalization experience (4 items). Normal-theory maximum-likelihood estimation was used. Model fit was evaluated using chi-square, the comparative fit index (CFI), Tucker–Lewis index (TLI), root mean square error of approximation (RMSEA), and standardized root mean square residual (SRMR), considered jointly rather than as inflexible pass–fail rules (Kline, 2023). The hypothesized four-factor model was compared with a three-factor model that combined cognition and practice and with a one-factor model that loaded all items on a single factor. Standardized factor loadings and latent factor correlations were also examined.

Three analytic decisions were made to improve the transparency of the results. First, because the measures were developed for this study, reliability and factor-structure evidence were reported before testing the substantive hypotheses. Second, moral cognition and moral practice were analyzed separately rather than combined into a single score, because the theory and the educational problem both require attention to the possible gap between knowing and doing. Third, the exploratory moderation analyses were treated as supplementary. They were used to check whether the main associations were stable across gender and grade, but they were not central to the hypothesized mechanism.

Mediation analyses were conducted PROCESS macro for SPSS, Version 4.1, Model 4, with 5000 bootstrap samples and 95% confidence intervals (Hayes, 2022). Bootstrap confidence intervals were used because estimates of indirect associations often have asymmetric sampling distributions. Exploratory moderation analyses using PROCESS Model 1 tested whether gender or grade moderated the associations between perceived reading ability and the two moral outcomes. Statistical significance was evaluated at *p* < 0.05. Because all focal variables were measured by self-reporting, common method variance cannot be ruled out; this issue was evaluated as an initial diagnostic check and is considered again in the limitations. To provide a broader assessment of internal consistency, McDonald’s omega was calculated alongside Cronbach’s alpha. Common method bias was evaluated in two stages. Harman’s single-factor test was retained as an initial diagnostic, after which an unmeasured latent method factor analysis was conducted. A correlated four-trait-factor measurement model was compared with a model that added an orthogonal latent method factor loading on all 18 focal self-report items. Model fit was evaluated using chi-square, CFI, TLI, RMSEA, and SRMR. Throughout the manuscript, PROCESS coefficients are therefore described as overall, conditional, and indirect associations rather than as causal effects.

## 3. Results

### 3.1. Preliminary Psychometric Analyses and Common Method Bias

The self-developed measures showed acceptable preliminary psychometric properties in the current sample (Table 1). EFA supported a one-factor solution for perceived classical Chinese text reading ability and a one-factor solution for internalization experience. The traditional moral scale showed a two-factor solution corresponding to moral cognition and moral practice. Cronbach’s alpha values were good for perceived reading ability, moral practice, and internalization experience. The moral cognition subscale showed modest internal consistency (Cronbach’s α = 0.641; McDonald’s ω = 0.681). The five retained items were intentionally designed to represent the five conceptually distinct Confucian virtues rather than interchangeable manifestations of a narrowly homogeneous construct. The subscale was therefore retained to preserve content coverage. A sensitivity analysis excluding the weakest retained item increased α to 0.726, but the associations and standardized regression coefficients involving perceived reading ability and internalization experience remained in the same direction and statistically significant. This indicated that the substantive conclusions were not dependent on that single item, although findings involving moral cognition should still be interpreted cautiously.

The hypothesized correlated four-factor CFA model provided an acceptable representation of the data, χ^2^(129) = 291.45, CFI = 0.937, TLI = 0.925, RMSEA = 0.064, and SRMR = 0.057. It fit significantly better than a three-factor model in which moral cognition and moral practice were combined, χ^2^(132) = 360.63, CFI = 0.911, TLI = 0.897, RMSEA = 0.076, and SRMR = 0.063, Δχ^2^(3) = 69.18, *p* < 0.001, and substantially better than a one-factor model, χ^2^(135) = 716.52, CFI = 0.774, TLI = 0.744, RMSEA = 0.119, and SRMR = 0.085, Δχ^2^(6) = 425.07, *p* < 0.001. Standardized loadings ranged from 0.704 to 0.882 for perceived reading ability, 0.144 to 0.759 for moral cognition, 0.583 to 0.748 for moral practice, and 0.771 to 0.863 for internalization experience. Latent factor correlations ranged from 0.548 to 0.758. These results provide preliminary support for distinguishing the four broader constructs. However, the low loading of one moral-cognition item, together with the modest reliability and average variance extracted for that subscale, indicates that its convergent validity remains limited and that findings involving moral cognition should be interpreted cautiously. Detailed moral-cognition item diagnostics and sensitivity analyses are provided in Tables S1 and S2 in the Supplementary Materials.

Harman’s single-factor test was first used as an initial diagnostic check for common method bias. An unrotated principal component analysis of all items extracted four factors with eigenvalues greater than 1, and the first factor explained 41.60% of the variance. Because this value slightly exceeded the commonly used 40% threshold, an unmeasured latent method factor analysis was conducted. The correlated four-trait-factor model showed an acceptable fit, χ^2^(129) = 291.45, CFI = 0.937, TLI = 0.925, RMSEA = 0.064, and SRMR = 0.057. Adding an orthogonal latent method factor improved model fit, χ^2^(111) = 187.12, CFI = 0.970, TLI = 0.959, RMSEA = 0.048, and SRMR = 0.038, Δχ^2^(18) = 104.33, *p* < 0.001, ΔCFI = 0.034. Thus, a method component was detectable. However, the method factor accounted for an average of approximately 4.4% of item variance, and the correlations among the four substantive trait factors remained stable after the method factor was included. These findings suggest that common method variance was present but was not sufficient to account fully for the substantive associations. The method-factor model comparison and latent trait correlations are reported in Tables S3 and S4 in the Supplementary Materials.

### 3.2. Descriptive Statistics and Correlations

Pearson correlations showed that perceived classical Chinese text reading ability was significantly and positively correlated with Confucian virtue cognition, Confucian virtue practice, and internalization experience. Moral cognition and moral practice were also significantly correlated with each other, and both were positively correlated with internalization experience (Table 2). These associations supported the expectation that students who reported stronger perceived reading ability also reported stronger moral understanding, more frequent moral practice, and deeper internalization experience.

### 3.3. Hierarchical Regression Analyses

Hierarchical regression analyses were conducted to examine whether perceived reading ability predicted the two moral outcomes after gender and grade were controlled. For moral practice, the demographic model was significant, R^2^ = 0.05, *F* = 5.50, *p* = 0.001. Compared with Grade 7 students, Grade 8 students (β = −0.24, *p* < 0.001) and Grade 9 students (β = −0.18, *p* = 0.005) reported lower levels of moral practice. Adding perceived reading ability significantly increased explained variance, ΔR^2^ = 0.20, *p* < 0.001, and perceived reading ability significantly predicted moral practice (β = 0.45, *p* < 0.001). When internalization experience was added, explained variance increased again, ΔR^2^ = 0.21, *p* < 0.001. In the final model, internalization experience significantly predicted moral practice (β = 0.61, *p* < 0.001), whereas the conditional regression coefficient for perceived reading ability was no longer significant (β = 0.07, *p* = 0.229).

For moral cognition, the demographic model was significant, R^2^ = 0.03, *F* = 3.35, *p* = 0.019. Grade 8 students reported lower moral cognition than Grade 7 students (β = −0.20, *p* = 0.003), whereas the Grade 9 contrast was not significant. Adding perceived reading ability significantly increased explained variance, ΔR^2^ = 0.22, *p* < 0.001, and perceived reading ability significantly predicted moral cognition (β = 0.48, *p* < 0.001). Adding internalization experience further increased explained variance, ΔR^2^ = 0.10, *p* < 0.001. In the final model, both perceived reading ability (β = 0.22, *p* < 0.001) and internalization experience (β = 0.42, *p* < 0.001) significantly predicted moral cognition, whereas gender and grade were no longer significant. Table 3 summarizes the regression models.

### 3.4. Cross-Sectional Mediation Analyses

PROCESS Model 4 was used to estimate indirect statistical associations through internalization experience between perceived classical Chinese text reading ability and the two moral outcomes. Gender and grade were controlled in both mediation models. Unstandardized coefficients are reported for the modeled paths; these paths represent associations rather than causal directions.

**Figure 1:**
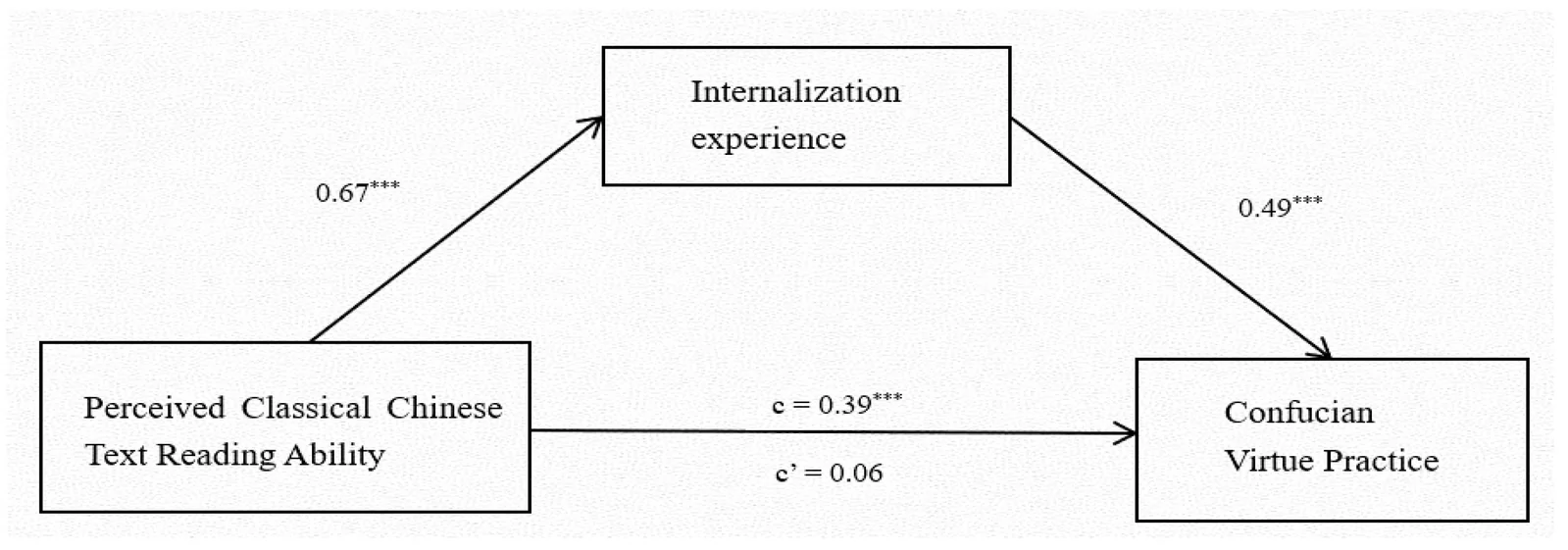
Cross-sectional mediation model for Confucian virtue practice. *** *p* < 0.001.

For Confucian virtue practice, perceived classical Chinese text reading ability significantly predicted internalization experience, B = 0.67, *p* < 0.001. Internalization experience also significantly predicted moral practice, B = 0.49, *p* < 0.001. The overall association between perceived reading ability and moral practice was significant, B = 0.39, *p* < 0.001. However, after internalization experience was included in the model, the conditional association between perceived reading ability and moral practice was no longer significant, B = 0.06, *p* = 0.214. The bootstrap analysis showed a significant indirect association, estimate = 0.33, Boot SE = 0.05, 95% CI [0.24, 0.43] (Figure 1). This confidence interval did not include zero. These results show a significant indirect association through internalization experience between perceived reading ability and moral practice, whereas the conditional association was nonsignificant.

**Figure 2:**
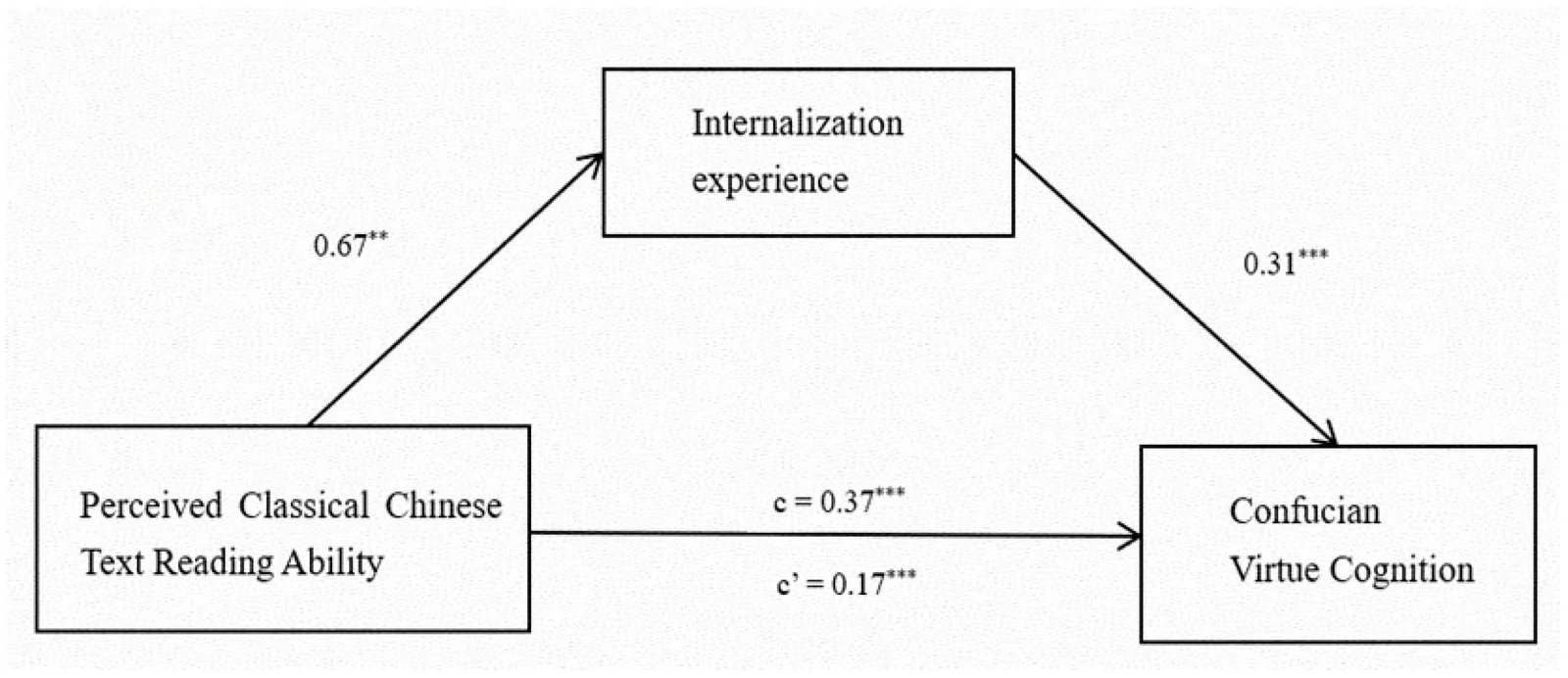
Cross-sectional mediation model for Confucian virtue cognition. *** *p* < 0.001, ** *p* < 0.01.

For Confucian virtue cognition, perceived classical Chinese text reading ability also significantly predicted internalization experience, B = 0.67, *p* < 0.001. Internalization experience significantly predicted moral cognition, B = 0.31, *p* < 0.001. The overall association between perceived reading ability and moral cognition was significant, B = 0.37, *p* < 0.001. After internalization experience was included in the model, the conditional association remained significant, B = 0.17, *p* < 0.001. The bootstrap analysis showed a significant indirect association, estimate = 0.20, Boot SE = 0.04, 95% CI [0.14, 0.28] (Figure 2). This confidence interval also did not include zero. These results show a significant indirect association through internalization experience between perceived reading ability and moral cognition, together with a significant remaining conditional association.

Taken together, the cross-sectional mediation results identify an indirect statistical association involving internalization experience between perceived classical Chinese text reading ability and students’ Confucian virtue cognition and practice. For moral practice, the conditional association of perceived reading ability was nonsignificant after internalization experience was included. This pattern does not establish temporal precedence or causal direction.

### 3.5. Supplementary Moderation Analyses

To further examine the role of demographic variables, exploratory moderation analyses were conducted using PROCESS Model 1. Perceived classical Chinese text reading ability was entered as the predictor, and moral cognition or moral practice was entered as the outcome. Gender and grade were tested separately as moderators. Because the grade groups were markedly unequal (Grade 7, *N* = 87; Grade 8, *N* = 176; Grade 9, *N* = 41), particularly with the relatively small Grade 9 subgroup, the exploratory grade-moderation results should be interpreted cautiously.

Gender did not significantly moderate the association between perceived classical Chinese text reading ability and moral cognition, *F*(1, 298) = 0.65, *p* = 0.420. It also did not moderate the association between perceived classical Chinese text reading ability and moral practice, *F*(1, 298) = 0.16, *p* = 0.685. Grade did not significantly moderate the association between perceived classical Chinese text reading ability and moral practice, *F*(2, 297) = 0.29, *p* = 0.747. The interaction between grade and perceived classical Chinese text reading ability in predicting moral cognition approached significance, *F*(2, 297) = 2.78, *p* = 0.064. This exploratory interaction did not reach the conventional significance level and therefore does not provide evidence of grade moderation. The possibility may be examined further using a larger and more balanced sample.

Overall, the results did not provide strong evidence that gender or grade played a stable moderating role in the relationships between perceived classical Chinese text reading ability and students’ traditional Confucian virtue cognition and practice.

## 4. Discussion

This study examined the relationships between Perceived Classical Chinese Text reading ability and junior secondary school students’ Confucian virtue cognition and moral practice. It also examined indirect statistical associations involving internalization experience in traditional cultural learning and explored possible demographic differences. The results showed that perceived classical Chinese text reading ability was significantly and positively associated with both Confucian virtue cognition and Confucian virtue practice. A significant indirect association through internalization experience was observed between perceived classical Chinese text reading ability and moral practice. For moral cognition, both an indirect association through internalization experience and a remaining conditional association were observed between perceived classical Chinese text reading ability and moral cognition. These cross-sectional patterns should not be interpreted as evidence of causal or temporal ordering. Supplementary analyses showed that gender did not have a stable moderating effect. No statistically significant grade moderation was detected, although one exploratory grade interaction approached significance.

A central contribution of these findings is that they clarify a cross-sectional pattern of associations among perceived classical Chinese text reading ability, internalization experience, and Confucian virtue cognition and practice. Previous discussions of classical Chinese education have often emphasized its cultural or normative importance, but such discussions do not always explain why some students may benefit more from traditional texts than others. The present findings suggest that perceived reading ability may be relevant because it reflects students’ perceived access to the moral meanings embedded in texts, but this cognitive access is not sufficient by itself, especially for behavioral outcomes. The association between perceived reading ability and moral practice was accompanied by a significant indirect association through internalization experience. Because the data were cross-sectional, this pattern is consistent with, but does not demonstrate, a developmental process. The observed pattern is instead consistent with the possibility that Confucian virtue cognition and practice involve both cognitive understanding and motivational internalization.

More specifically, the contribution is integrative rather than the proposal of a new universal stage model of moral development. Reading-comprehension theory explains how students may gain cognitive access to the explicit and implicit meanings of culturally embedded texts; moral-education research identifies the distinction between understanding a virtue and reporting its enactment; and self-determination theory explains why values experienced as meaningful and self-endorsed may be more closely associated with reported practice. Bringing these perspectives together clarifies that text comprehension and value internalization are analytically distinct components of the proposed associational model. The present cross-sectional findings offer preliminary support for this integrated account, while longitudinal and experimental research is required to test its temporal and causal propositions.

Consistent with this construct definition, the two outcomes are interpreted as concurrent domains of Confucian virtue acquisition rather than as evidence of virtue acquisition. The cross-sectional design cannot establish a developmental transition from virtue cognition to virtue practice or change in either domain over time.

### 4.1. Perceived Classical Chinese Text Reading Ability and Confucian Virtue Cognition and Practice

The present study first confirmed a significant association between perceived classical Chinese text reading ability and students’ traditional Confucian virtue cognition and practice. Correlation results showed that perceived reading ability was positively related to both Confucian virtue cognition and Confucian virtue practice. Hierarchical regression analyses further showed that perceived reading ability still significantly predicted the two moral outcomes after gender and grade were controlled.

These cross-sectional findings indicate that perceived classical Chinese text reading ability was associated with students’ language comprehension and text-processing skills, as well as with Confucian virtue cognition and practice. Students reporting stronger perceived reading ability also reported greater understanding of the moral meanings, value judgments, and behavioral norms embedded in traditional cultural texts. These concurrent associations do not determine whether perceived reading ability precedes moral meaning-making or develops alongside it.

### 4.2. Indirect Associations Involving Internalization Experience

Another important finding is the statistically significant indirect association involving internalization experience between perceived classical Chinese text reading ability and traditional Confucian virtue cognition and practice. The cross-sectional patterns differed between moral cognition and moral practice. In the model for moral practice, the overall association between perceived reading ability and moral practice was significant, but the conditional association was nonsignificant after internalization experience was included. In the model predicting moral cognition, both the conditional and indirect associations remained significant.

This difference is theoretically informative, but it remains associational. Stronger perceived classical Chinese text reading ability was associated with students’ reported understanding of the moral concepts, value judgments, and behavioral norms contained in texts. This co-occurrence may be relevant to moral cognition, although its direction cannot be determined. Moral practice was likewise associated with personally meaningful and self-endorsed values. This pattern is consistent with self-determination theory, which emphasizes that external values are more likely to guide stable behavior when they are understood, accepted, and integrated into the self (Deci & Ryan, 2000; Ryan & Deci, 2017).

Conceptually, the aggregate association involving internalization experience can be understood in terms of these complementary experiences: perceived meaningfulness connects textual content with students’ lives; value identification reflects personal endorsement of the virtues; emotional engagement gives the moral content affective salience; and self-integration connects the endorsed values with self-relevant standards for action. However, because the four items formed a single overall scale and the data were cross-sectional, the present findings neither demonstrate a sequential four-stage internalization process nor isolate the unique statistical contribution of any one facet. The results should therefore be interpreted as evidence of an overall association involving internalization experience rather than as a test of separate facet-level mechanisms.

The findings are also consistent with recent Confucian virtue cognition and practice research suggesting that moral judgment, emotional engagement, moral identity, and prosocial behavior are closely connected (Cameron et al., 2022; L. Wang et al., 2025). The present study adds to this literature by identifying significant cross-sectional associations involving internalization experience, culturally embedded text comprehension, moral cognition, and moral practice. These patterns identify internalization experience as a potentially relevant correlate of both moral knowing and moral doing, without establishing that it causes either outcome.

### 4.3. Supplementary Analyses of Demographic Variables

This study also conducted supplementary analyses of grade and gender. The results showed that gender did not have a stable moderating effect. This suggests that the positive associations between perceived classical Chinese text reading ability and Confucian virtue cognition and practice were generally similar for boys and girls.

Grade also did not significantly moderate the relationship between perceived classical Chinese text reading ability and moral practice. Only the interaction between grade and perceived reading ability in predicting moral cognition approached significance. This result suggests that the strength of the association between perceived reading ability and moral cognition may differ somewhat across grades, but this effect was not strong enough to support a clear conclusion. Future studies should use larger samples and longitudinal designs to further examine how demographic factors may shape the relationship between traditional cultural learning and adolescent Confucian virtue cognition and practice.

### 4.4. Educational Implications

The findings have practical implications for traditional cultural education and moral education in schools. First, teaching should place more emphasis on deep interpretation and value analysis. Classical Chinese learning should not stop at word translation or sentence explanation. Teachers can guide students to analyze characters’ actions, ethical conflicts, and narrative logic. This can help students identify the value norms embedded in texts and make traditional cultural learning a process that combines cognitive training with reflection on and application of Confucian virtues.

Second, teachers should make internalization experience an important goal of instructional design. Students need opportunities to connect traditional cultural values with their own lives. Classroom activities such as situational discussion, real-life connection, role taking, reflective writing, and value clarification may help students experience traditional cultural content as meaningful, personally relevant, and emotionally engaging. For example, after reading a classical passage related to integrity, students could discuss situations in which honesty conflicts with peer pressure or personal benefit. After reading a passage related to benevolence or propriety, they could reflect on interpersonal conflicts in school life and consider how traditional virtues might inform their responses.

To translate these principles into classroom practice, teachers could organize a classical Chinese lesson around a five-step sequence. First, before reading, teachers could provide focused support for difficult vocabulary, syntax, and historical context and pose a value-oriented guiding question. Second, during close reading, students could complete a character–choice–consequence–virtue organizer that requires them to cite textual evidence rather than merely provide a modern Chinese translation. Third, teachers could use a structured moral-dilemma discussion in which students compare alternative actions, identify possible conflicts among virtues, and justify their interpretations. Fourth, perspective-taking or role-play could connect the textual dilemma to a realistic situation involving peers, family, or school life. Fifth, students could complete a brief reflective journal or personal action plan identifying a situation in which the focal virtue might be enacted. This sequence connects linguistic comprehension, value interpretation, emotional engagement, and self-integration without reducing classical Chinese instruction to moral exhortation.

Activities can also be tailored to the five Confucian virtues. For benevolence, students might analyze a character’s emotions and needs and design a supportive response; for righteousness, they might debate a fair response to a conflict of interest; for propriety, they might revise a disrespectful interaction to preserve both respect and appropriate boundaries; for wisdom, they might compare the evidence and consequences behind different choices; and for integrity, they might discuss dilemmas involving honesty, promises, or peer pressure. Teachers should use open-ended questions and permit reasoned disagreement so that students practice interpretation rather than reproduce a predetermined moral answer. Formative assessment could combine text-based interpretation, reflective reasoning, and scenario-based application. Brief rubrics could evaluate the use of textual evidence, perspective-taking, clarity of value reasoning, and feasibility of proposed actions, while journals, exit tickets, teacher observations, and peer-discussion records could document engagement without treating conformity as proof of virtue acquisition. Because the present study was cross-sectional, these measures should be regarded as theory-informed instructional proposals whose effectiveness requires evaluation through classroom intervention studies.

Third, teaching should pay attention to differences among student groups. Although gender and grade did not show stable moderating effects in this study, students may still differ in learning engagement, value experience, and developmental needs. Students in higher grades may face stronger academic pressure, and their learning of classical texts may become more examination-oriented. Teachers should therefore create space for meaning exploration and value reflection even in test-driven contexts.

### 4.5. Limitations and Future Directions

Several limitations should be noted. First, this study used a cross-sectional design. The results showed significant relationships among perceived classical Chinese text reading ability, internalization experience, moral cognition, and moral practice; however, the mediation models establish neither temporal precedence nor causality. Reverse or reciprocal relationships are also plausible. Future research should use multiwave longitudinal designs to test temporal ordering and changes over time. Experimental or intervention studies are also needed to determine whether changes in students’ perceived classical Chinese text reading ability precede changes in internalization experience and Confucian virtue cognition and practice.

Second, perceived classical Chinese text reading ability was assessed using a brief four-item self-report scale rather than an objective reading-performance test. Consequently, the measure reflected adolescents’ subjective perceptions of their reading ability and might not accurately represent their actual literacy competence. Self-reports may also be affected by social desirability, inaccurate self-perception, and common method bias. Future studies should combine self-report measures with objective classical Chinese reading-performance tests, such as true/false or multiple-choice questions assessing vocabulary comprehension, contextual translation, main-idea identification, inferential comprehension, and the analysis of characters’ moral qualities. Such multi-method assessment would help validate the robustness of the present findings. Future research should also incorporate other data sources, such as teacher ratings, peer ratings, behavioral tasks, situational judgment tests, and classroom observations.

Because data collection had been completed before this diagnostic analysis was conducted, a theoretically unrelated marker variable and temporal or source separation could not be introduced retrospectively. Future studies should address common method variance prospectively by separating predictor and outcome measurements in time, obtaining outcome data from teachers or behavioral tasks, protecting respondent anonymity, counterbalancing item order where appropriate, and including a theoretically unrelated marker variable.

Third, the measures used in this study were self-developed and brief. Although the four-factor CFA supported the distinction among the broader constructs, it was conducted using the same sample as the exploratory analyses and therefore does not constitute independent cross-validation. Moreover, each specific reading content area, Confucian virtue, and internalization facet was represented by only one item. The present study treated these items as content indicators of broader overall constructs and did not interpret them as reliable single-item subscales. Nevertheless, this design limits content depth and prevents facet-specific reliability and validity assessment. The moral cognition subscale also showed modest internal consistency, low average variance extracted, and one weak CFA loading. Future research should develop at least three items for each facet intended to be interpreted separately, conduct cognitive interviews and pilot testing, use independent samples for EFA and CFA, and evaluate convergent validity, discriminant validity, criterion validity, test–retest reliability, and measurement invariance across grades, regions, and school types.

Fourth, the sample was drawn from three junior secondary schools in Ningbo, China. The schools were purposively selected to vary in administrative location and school type, but the design did not use probability sampling and cannot establish representativeness. In addition, family socioeconomic indicators such as parental education, occupation, and household income were not collected, so variation in family background could not be described or controlled. Future studies should collect these variables and use larger, cross-regional samples, preferably with proportional stratified sampling across school location, school type, grade, and socioeconomic strata. Finally, future research could compare traditional translation-based instruction with instruction that includes discussion, reflection, and value-internalization activities. These sampling constraints limit the generalizability of the findings in several ways. Regionally, the three participating schools were located in Ningbo, a coastal city in eastern China, and may not represent adolescents studying in inland, western, rural, or ethnic-minority settings, where educational resources, teacher preparation, exposure to classical Chinese texts, and curriculum implementation may differ. Culturally, familiarity with Confucian traditions and the meanings or salience assigned to the five virtues may vary across regions and communities; therefore, the strength and even the form of the observed associations should not be assumed to be invariant across China. Socio-economically, differences in parental education, household income, access to books or tutoring, and school resources may shape both students’ perceived reading ability and their opportunities to understand and enact Confucian virtues. Generalization beyond China requires still greater caution because classical Chinese texts are embedded in a specific linguistic, historical, and Confucian educational tradition. In other cultural settings, analogous associations may depend on locally valued texts, moral frameworks, and practices of value socialization. Accordingly, the present findings should be regarded as context-specific preliminary evidence rather than as nationally or globally representative. Future multisite and cross-cultural studies should sample diverse regions and socioeconomic strata and establish measurement equivalence before comparing the associations across cultural groups.

The grade distribution was also imbalanced (Grade 7, *N* = 87; Grade 8, *N* = 176; Grade 9, *N* = 41), leaving the Grade 9 subgroup particularly small. This imbalance limited the statistical power and precision of the exploratory grade-moderation analyses. Future research should employ proportional stratified sampling by grade to obtain more balanced subsamples and more reliable estimates of grade differences.

## 5. Conclusions

This study found that junior secondary school students’ perceived classical Chinese text reading ability was significantly and positively associated with both Confucian virtue cognition and Confucian virtue practice. Cross-sectional mediation analysis identified a significant indirect association through internalization experience between perceived reading ability and moral cognition, as well as between perceived reading ability and moral practice; however, these patterns do not establish causal or temporal ordering. Supplementary analyses showed that gender did not have a stable moderating effect, and no statistically significant grade moderation was detected, although one exploratory grade interaction approached significance. Overall, perceived classical Chinese text reading ability was associated with adolescents’ Confucian virtue cognition and practice, with stronger internalization experience statistically accompanying stronger moral outcomes.

Accordingly, the findings concern cross-sectional differences in Confucian virtue acquisition, operationalized as Confucian virtue cognition and practice, rather than longitudinal moral development or a transition from moral understanding to moral action over time.

## Figures and Tables

**Table 1:** Preliminary psychometric evidence for the study measures.

Measure	Items	KMO	Variance Explained	Cronbach’s Alpha	McDonald’s Omega
Perceived reading ability	4	0.824	72.61%	0.871	0.876
Confucian virtue cognition	5	0.854	51.41%	0.641	0.681
Confucian virtue practice	5	0.854	51.41%	0.794	0.806
Internalization experience	4	0.826	73.24%	0.878	0.879

Note. KMO = Kaiser–Meyer–Olkin measure of sampling adequacy. For all exploratory factor analyses, Bartlett’s test of sphericity was significant at *p* < 0.001. The moral cognition and moral practice rows refer to the same two-factor EFA of the traditional moral scale after one weak item was removed. McDonald’s omega was estimated from a one-factor measurement model for each subscale.

**Table 2:** Descriptive statistics and correlations among the main variables.

Variable	*M*	*SD*	1	2	3	4
1. Perceived reading ability	4.81	1.12				
2. Confucian virtue cognition	5.34	0.86	0.50 ***			
3. Confucian virtue practice	5.33	0.96	0.48 ***	0.57 ***		
4. Internalization experience	5.09	1.19	0.65 ***	0.57 ***	0.67 ***	

Note. *N* = 304. *** *p* < 0.001.

**Table 3:** Hierarchical regression models predicting virtue cognition and virtue practice.

Outcome	Predictor	Model 1 β	Model 2 β	Model 3 β
Confucian virtue cognition	Gender	0.05	0.04	−0.004
	Grade 8 vs. Grade 7	−0.20 **	−0.08	−0.05
	Grade 9 vs. Grade 7	−0.06	0.02	0.04
	Perceived reading ability		0.48 ***	0.22 ***
	Internalization experience			0.42 ***
	R	0.18	0.51	0.60
	R^2^	0.03	0.26	0.36
	ΔR^2^	0.03	0.22 ***	0.10 ***
	*F*	3.35 *	25.59 ***	32.82 ***
Confucian virtue practice	Gender	0.06	0.04	−0.02
	Grade 8 vs. Grade 7	−0.24 ***	−0.13 *	−0.09
	Grade 9 vs. Grade 7	−0.18 **	−0.10	−0.08
	Perceived reading ability		0.45 ***	0.07
	Internalization experience			0.61 ***
	R	0.23	0.50	0.68
	R^2^	0.05	0.25	0.46
	ΔR^2^	0.05	0.20 ***	0.21 ***
	*F*	5.50 **	24.58 ***	49.90 ***

Note. Standardized regression coefficients are reported for predictors. Grade 7 was the reference category. * *p* < 0.05. ** *p* < 0.01. *** *p* < 0.001.

## Data Availability

The datasets generated and/or analyzed during the current study are available from the corresponding author on reasonable request. The study materials, including the self-developed measures used in this study, are also available from the corresponding author on reasonable request. The deidentified data, study materials, and analysis code are available from the corresponding author upon reasonable request, subject to applicable ethical and privacy restrictions.

## Supplementary Materials

The following supporting information can be downloaded at: https://www.mdpi.com/article/10.3390/bs16091659/s1, Table S1: Item content and item-level psychometric evidence for the final five-item Confucian virtue cognition subscale; Table S2: Sensitivity analysis comparing the five-item moral-cognition score with a four-item score excluding Item 4; Table S3: Fit of the correlated four-factor measurement model and the model including an unmeasured latent method factor; Table S4: Latent correlations before and after inclusion of the unmeasured latent method factor.
