# Supplementary material for: Associations of Perceived Classical Chinese Text Reading Ability with Confucian Virtue Cognition and Practice Among Chinese Adolescents: The Indirect Role of Internalization Experience
Source: Behav Sci (Basel). 2026 Sep 16;16(9):1659. doi: 10.3390/bs16091659 (PMC13603912; doi:10.3390/bs16091659)
Supplement: Supplementary file 1 [file behavsci-16-01659-s001.zip › behavsci-4408733-supplementary.pdf]

Supplementary Materials

Associations of Perceived Classical Chinese Text Reading Ability with Confucian Virtue Cognition and Practice Among Chinese Adolescents: The Indirect Role of Internalization Experience

All analyses reported below used the complete sample (N = 304). No focal item values were missing. The supplementary analyses were conducted to document item-level evidence, evaluate sensitivity to the weak moral-cognition item, and assess common method variance.

Table S1. Item content and item-level psychometric evidence for the final five-item Confucian virtue cognition subscale.

Panel A. Item wording

Item	Virtue	English wording	Original Chinese wording
Item 1	Benevolence (ren)	I believe that 'the benevolent love others' still provides guidance in modern society.	我认为“仁者爱人”在现代社会仍有指导意义。
Item 2	Righteousness (yi)	When facing conflicts of interest, I would give priority to the principle of righteousness.	面对利益冲突时，我会优先考虑“义”的原则。
Item 3	Propriety (li)	Observing norms of propriety is an important expression of personal cultivation.	遵守礼仪规范是个人修养的重要体现。
Item 4	Wisdom (zhi)	I believe that wisdom is more important than knowledge.	我认为智慧比知识更重要。
Item 5	Integrity (xin)	One should uphold integrity at all times.	无论何时都应坚守诚信。

Panel B. Item diagnostics

Item	Standardized factor loading	Corrected item-total correlation	Cronbach's α if item deleted
Item 1	0.759	0.540	0.516
Item 2	0.686	0.515	0.525
Item 3	0.620	0.514	0.538
Item 4	0.144	0.098	0.726
Item 5	0.483	0.378	0.597

Note. Responses ranged from 1 (completely disagree) to 7 (completely agree). English wording is a reporting translation of the original Chinese items. For the five-item subscale, Cronbach's α = 0.641, McDonald's ω = 0.681, composite reliability (CR) = 0.681, and average variance extracted (AVE) = 0.337. Standardized loadings are from the correlated four-factor CFA. Item 4 showed the weakest evidence (corrected item-total correlation = 0.098; α if deleted = 0.726). A sixth reverse-keyed item ('Occasionally lying is acceptable') had been removed during the initial item-analysis stage and was not included in the final five-item score.

Table S2. Sensitivity analysis comparing the five-item moral-cognition score with a four-item score excluding Item 4.

Panel A. Reliability and zero-order associations

Statistic	Five-item estimate	p	Four-item estimate	p
Cronbach's α	0.641	-	0.726	-
Perceived reading ability with moral cognition (r)	0.495	< 0.001	0.484	< 0.001
Internalization experience with moral cognition (r)	0.566	< 0.001	0.584	< 0.001

Panel B. Regression coefficients

Path	B	SE	β	p	B	SE	β	p
a: Reading ability -> internalization experience	0.670	0.047	0.634	< 0.001	0.670	0.047	0.634	< 0.001
c: Overall reading-ability association with moral cognition	0.372	0.039	0.483	< 0.001	0.414	0.045	0.472	< 0.001
c': Conditional reading-ability association with moral cognition	0.167	0.047	0.217	< 0.001	0.156	0.054	0.177	0.004
b: Conditional internalization association with moral cognition	0.306	0.045	0.420	< 0.001	0.386	0.051	0.464	< 0.001

Columns 2-5: five-item scoring; columns 6-9: four-item scoring.

Panel C. Bootstrap indirect association

Cognition scoring	Indirect B	Boot SE	95% CI lower	95% CI upper	Final R^2
Five-item scoring	0.205	0.036	0.140	0.280	0.355
Four-item scoring	0.258	0.040	0.183	0.342	0.369

Note. The four-item score excludes the final scale's Item 4 ('I believe that wisdom is more important than knowledge'). Regression models controlled for gender and grade (Grade 7 reference; Grade 8 and Grade 9 dummy variables). B denotes an unstandardized coefficient and β denotes a standardized coefficient. Indirect-association confidence intervals were based on 5,000 percentile bootstrap samples. The direction, magnitude, and statistical significance of the focal associations were substantively unchanged after Item 4 was excluded.

Table S3. Fit of the correlated four-factor measurement model and the model including an unmeasured latent method factor.

Model	χ^2	df	CFI	TLI	RMSEA	SRMR
Correlated four-factor model	291.45	129	0.937	0.925	0.064	0.057
Four-factor + orthogonal method-factor model	187.12	111	0.970	0.959	0.048	0.038

Note. N = 304; 18 focal self-report items. The method factor was specified as orthogonal to the four trait factors. Relative to the four-factor model, adding the method factor yielded $\Delta\chi^2(18) = 104.33$, $p < 0.001$, ΔCFI = 0.034, ΔRMSEA = -0.017, and ΔSRMR = -0.018. The average variance attributable to the method factor was approximately 4.4%.

Table S4. Latent correlations before and after inclusion of the unmeasured latent method factor.

Latent variable pair	Four-factor model r	Method-factor model r	Change (Δr)	Absolute change
Perceived reading ability - Confucian virtue cognition	0.609	0.628	+0.018	0.018
Perceived reading ability - Confucian virtue practice	0.548	0.576	+0.028	0.028
Perceived reading ability - Internalization experience	0.725	0.723	-0.002	0.002
Confucian virtue cognition - Confucian virtue practice	0.730	0.701	-0.030	0.030
Confucian virtue cognition - Internalization experience	0.732	0.760	+0.028	0.028
Confucian virtue practice - Internalization experience	0.758	0.806	+0.048	0.048

Note. Change was calculated as the method-factor-model correlation minus the corresponding four-factor-model correlation. Absolute changes ranged from 0.002 to 0.048; thus, all six latent correlations changed by less than 0.05 after the method factor was included.
